# Supplementary material for: Ploidy dynamics in aphid host cells harboring bacterial symbionts
Source: Sci Rep. 2022 Jun 1;12:9111. doi: 10.1038/s41598-022-12836-8 (PMC9159990; doi:10.1038/s41598-022-12836-8)
Supplement: Supplementary file 1 — Supplementary Information. [file 41598_2022_12836_MOESM1_ESM.docx]

Original Research Article

**Ploidy dynamics in aphid host cells harboring bacterial symbionts**

Supplementary Information

Tomonari NOZAKI^1*^, Shuji SHIGENOBU^1,2^

*^1^Laboratory of Evolutionary Genomics, National Institute for Basic Biology, 38 Nishigonaka, Myodaiji, Okazaki, Aichi 444-8585, Japan*

*^2^Department of Basic Biology, School of Life Science, The Graduate University for Advanced Studies (SOKENDAI), 38 Nishigonaka, Myodaiji, Okazaki, Aichi 444-8585 Japan*

^*^*Correspondence*:

Tomonari Nozaki, Laboratory of Evolutionary Genomics, National Institute for Basic Biology, Okazaki, Aichi 444‐8585, Japan

Telephone: +81-564-55-7670

Email: nozaki.t@nibb.ac.jp, tomonari64.1.8@gmail.comSI Methods

Productivity and developmental schemes of female aphids

To compare the productivity and longevity between viviparous and oviparous aphids, newly molted adults were chosen and separately reared on the leaves of the bean plants, *Vicia faba*. Both females were apterous. Oviparous females were reared with young males from the same generation, allowing for free mating. These females were checked daily, and both the number of larviposition (the number of nymphs produced) by viviparous females and oviposition (the number of eggs produced) by oviparous females were recorded. The day of death was also recorded. Viviparous adults were maintained at 16 °C under a long-day photoperiod (16 h light:8 h dark), the same condition as the source populations. Oviparous adults were placed at 15 °C under a short-day condition (8 h light:16 h dark). All nymphs and eggs laid were removed immediately after the daily observation to avoid crowded conditions, which can influence female development ^[1]^ and induce oocyte/embryo resorption ^[2]^. To elucidate the developmental scheme (duration of each stage at 16 °C) of viviparous females, newly larviposited (first instar) nymphs were reared separately and checked daily to record the day of molting.

We compared the total number of larvae and eggs laid during the entire life of insects using a generalized linear mixed-effect model (GLMM) with a Poisson error distribution and log link function between viviparous and oviparous females. In this analysis, the types of females were treated as fixed effects, and individuals were included as a random effect. Longevity (= adult lifetime) of both types of females was also compared. In this model, the types of females were treated as a fixed effect, and individuals were included in a random effect. Note that the interaction between female types and their lifespan was analyzed but not significant (see Results).

Supplementary Figures


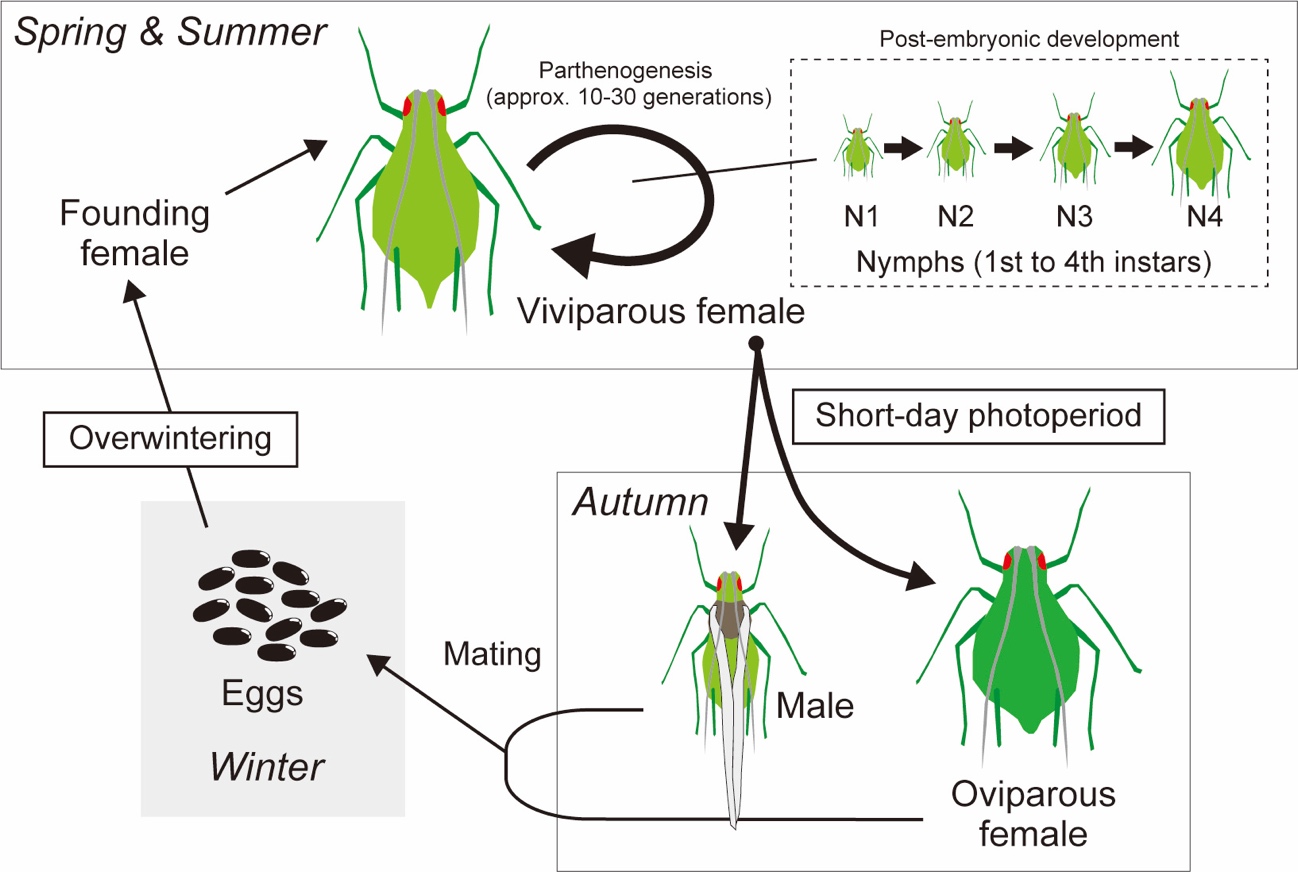


**Figure S1.** The life cycle and polymorphism of pea-aphids. In the field, viviparous females are observed from spring to summer on their host plants and rapidly reproduce through viviparous parthenogenesis. N1-4 represents the first to fourth instar nymphs. Larviposited nymphs grow up to adults after molting four times. Oviparous females and males emerge in autumn; they are induced by short-day photoperiod. Oviparous females mate with males and produce eggs (sexual reproduction). All aphid morphs harbor *B. aphidicola* inside of their bacteriocytes. Illustrations were drawn according to Ogawa & Miura^[30]^.


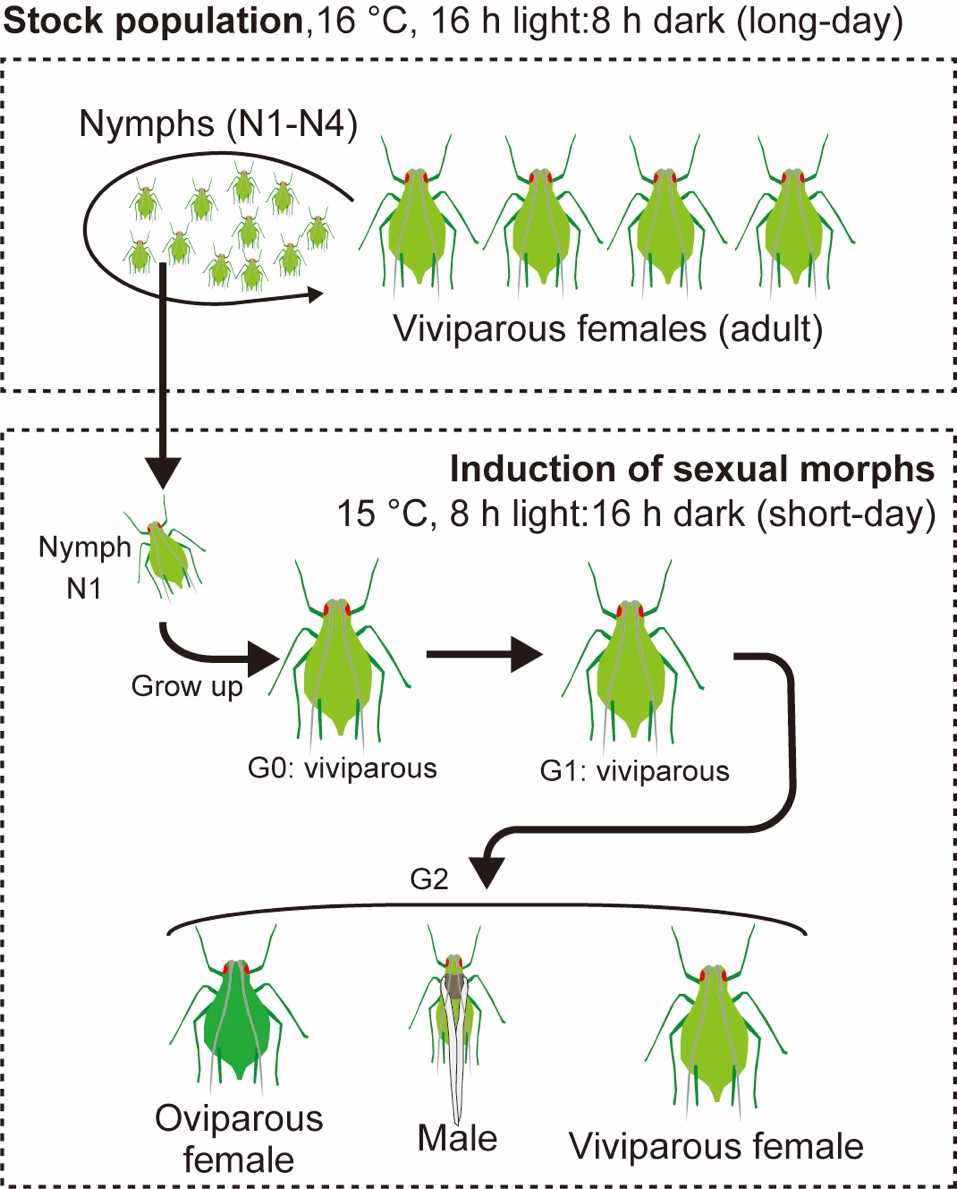


**Figure S2.** Detailed experimental procedure to induce males and oviparous females in the ApL strain. In the stock population, viviparous insects were maintained on young, broad bean plants (*Vicia faba* L.) under 16 °C, a photoperiod of 16 h light:8 h dark (long-day conditions). First instar nymphs isolated from stock populations (G0 insects) were reared on the broad bean plants, under 15 °C, 8 h light:16 h dark (short-day condition). G0 offspring (G1 insects) parthenogenetically produce sexual morphs (G2, Oviparous females and males). In strain ApL, G1 aphids also produce few viviparous females.


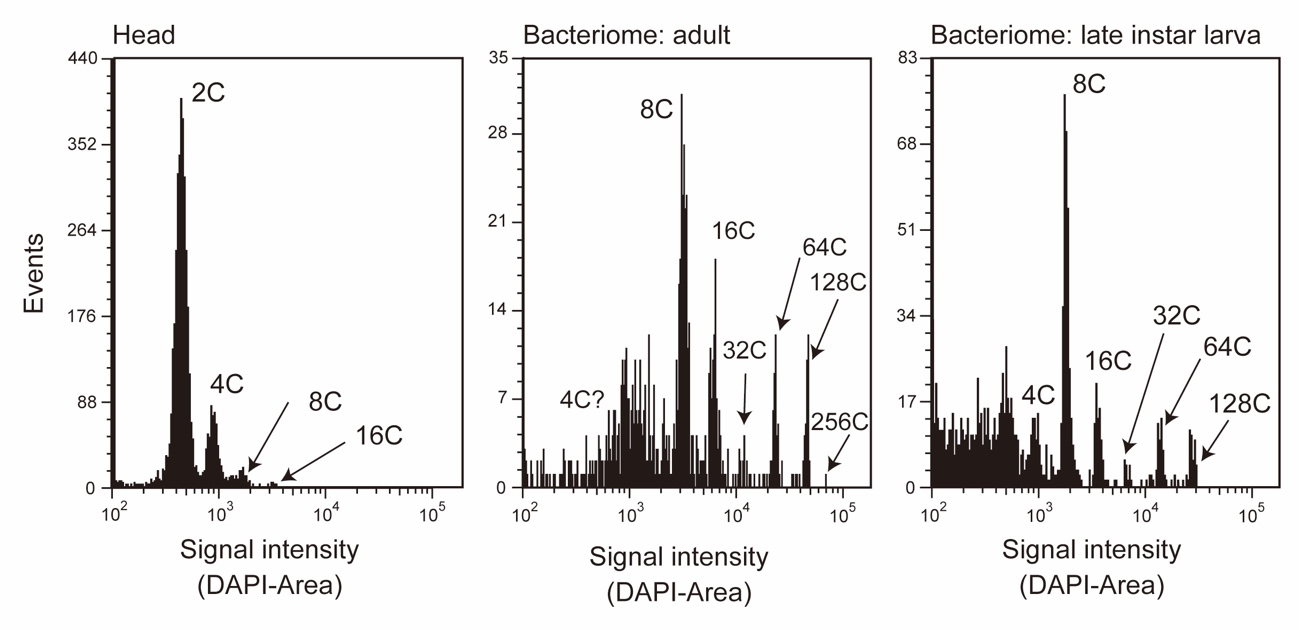


**Figure S3**. Examples of nuclear DNA content analysis by flow cytometry. The first peak in the head sample corresponds to the population of 2C-DNA nuclei, which was preliminarily determined by analysis of sperm cells (haploid; 1C) of males. “C” means haploid genome size, for example, 2C = diploid and 8C = octoploid. Analysis on bacteriome of adults and late instar nymphs revealed that this organ consisted of at most 256C cells, while cell types could not be identified.


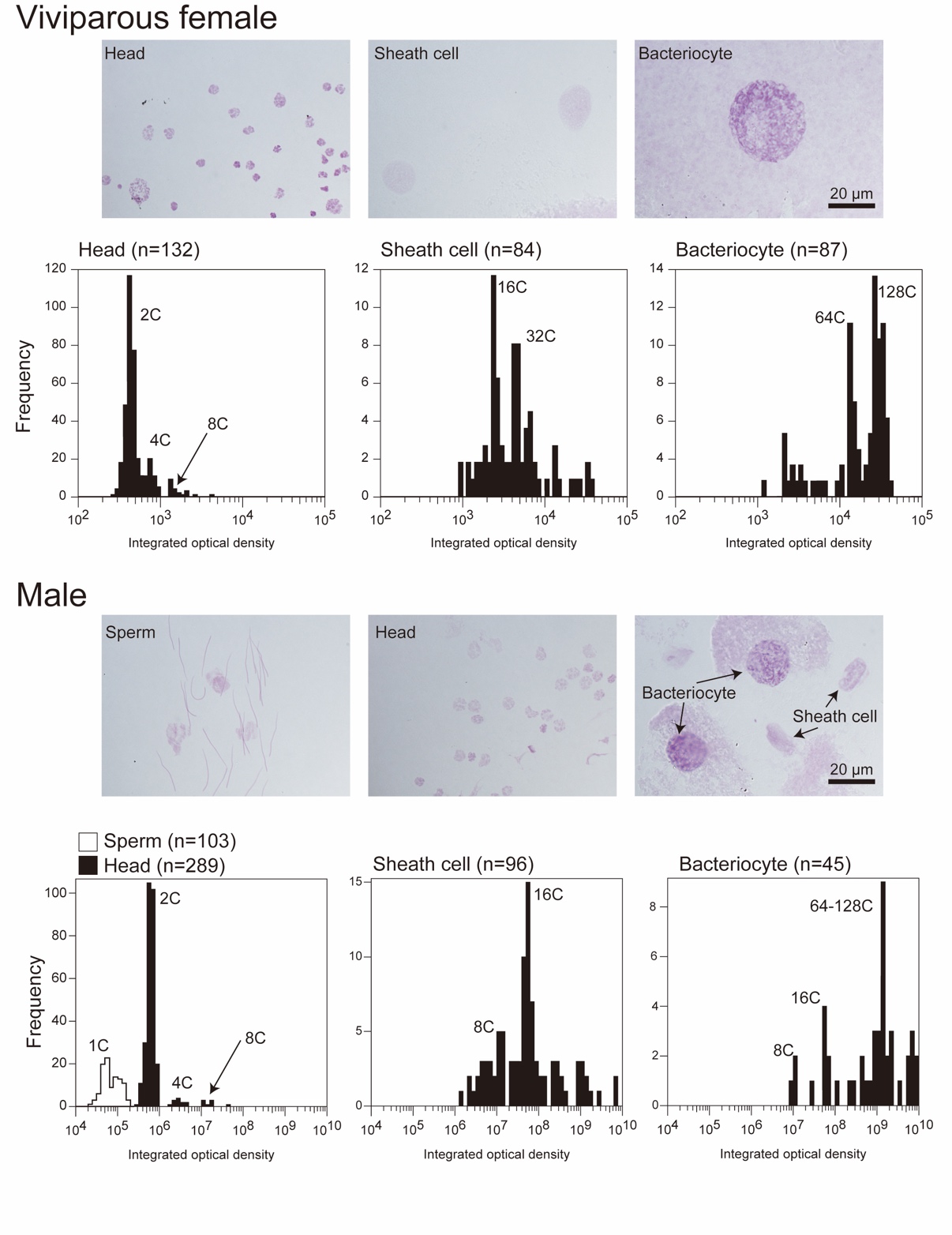


**Figure S4**. Examples of Feulgen-stained nuclei from different types of cells (head, sheath ell, bacteriocyte and sperm) and nuclear DNA content analysis by Feulgen densitometry. The first peak in the head sample corresponds to the population of 2C-DNA nuclei, which was determined by analysis of sperm cells (haploid; 1C) of males. Analysis of the bacteriome cells of adult viviparous aphids demonstrated that these bacteriocytes and sheath cells mainly consist of 64C and 128C, and 16C and 32C cells, respectively. However, each peak was not clearly separated, and the cell numbers were small; at most 32 bacteriocytes could be obtained from one individual. Data from three individuals were pooled and presented.


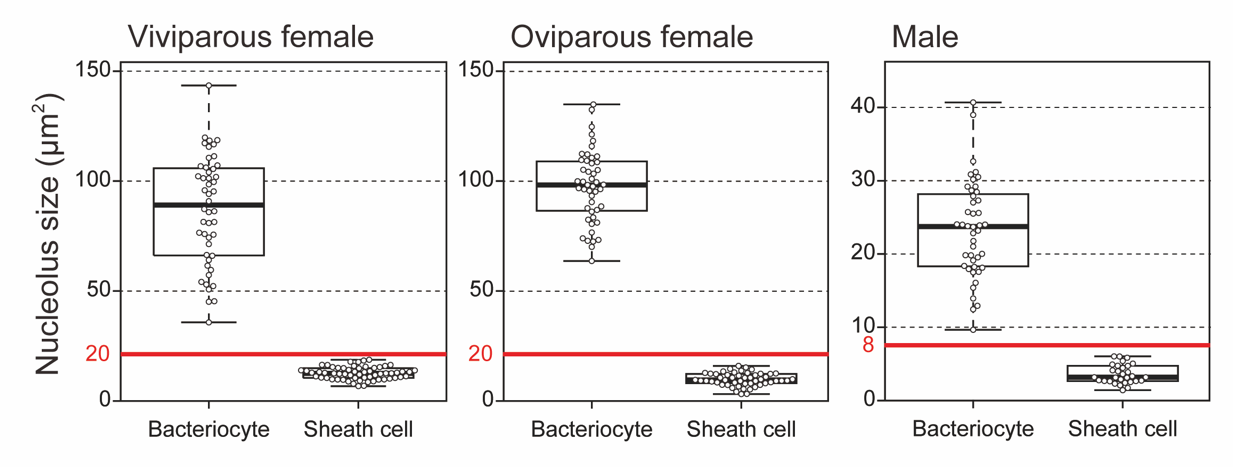


**Figure S5.** Nucleolus size distribution in three types of adults: viviparous females, oviparous females, and males. The size of nucleolus was significantly different between bacteriocytes and sheath cells, regardless of aphid morphs (LM with type II test, *p* < 0.001 in all). There was no overlap in nucleolus sizes between the two types of cells. Red bars indicate the “threshold” used in the image-based fluorometry (see Material and methods).


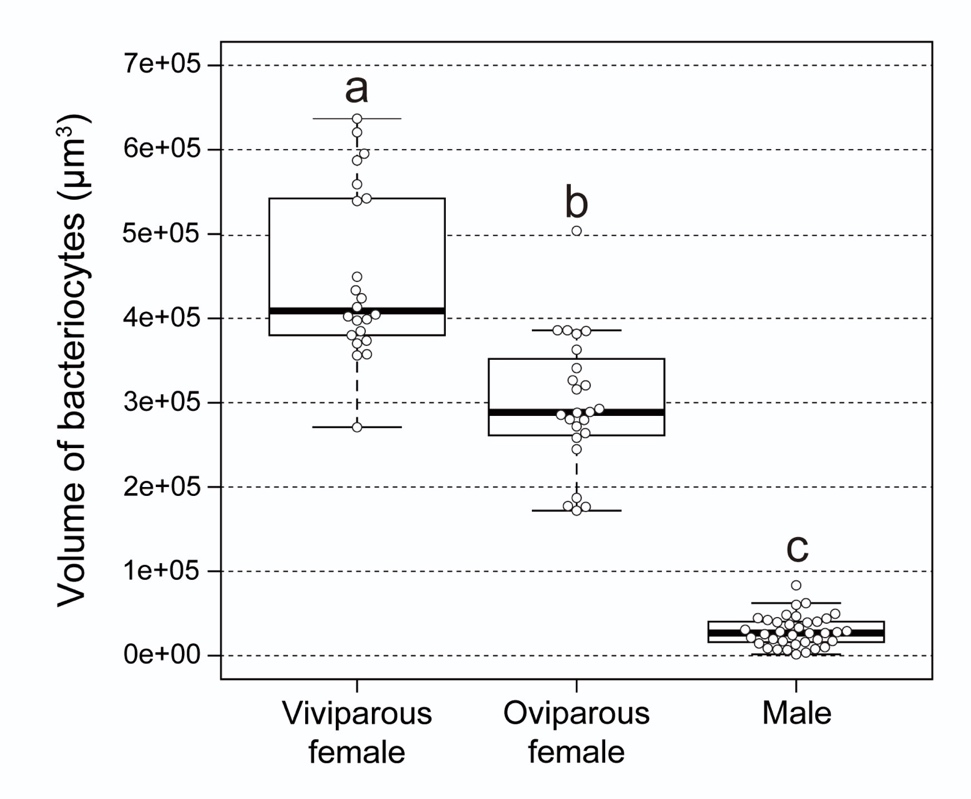


**Figure S6.** Cell volume distribution of bacteriocytes in each aphid morph. The volume of bacteriocytes was estimated based on the data from confocal microscopy (see Material and methods). Results are displayed as boxplots where central bold lines represent the medians, boxes comprise the 25–75 percentiles, and whiskers denote the range. The size of bacteriocytes was significantly different among aphid morphs [viviparous females; 449847.05 ± 21583.0 (mean ± SEM) μm^3^, *n* = 22, oviparous females; 298989.9 ± 16196.6 μm^3^, *n* = 24, and males; 29020.35 ± 3001.6 μm^3^, *n* = 37] (LM with type II test, *p* < 0.001). Different letters indicate significant differences (Tukey’s test, *p* < 0.05).


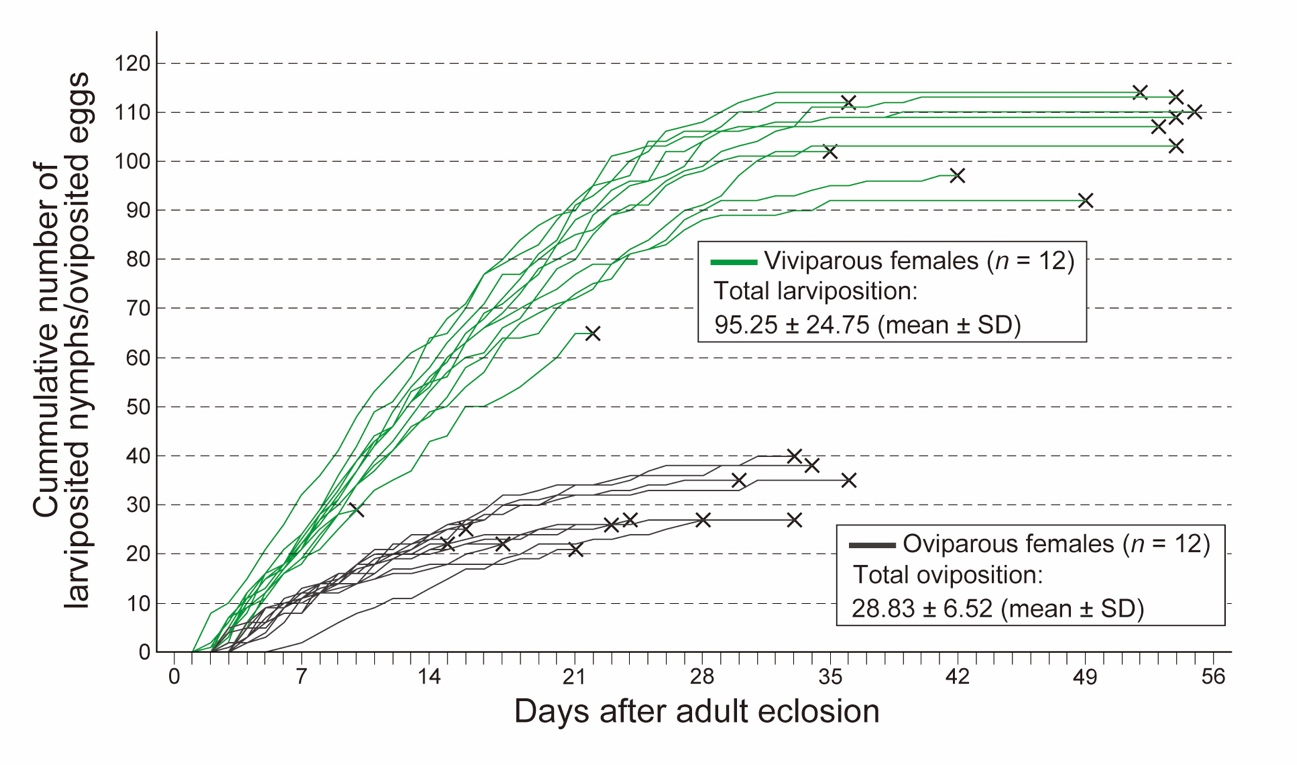


**Figure S7.** Reproduction activity in both types of aphid females. Viviparous aphids start larviposition several days after adult eclosion and reproduce actively during approximately 3 weeks. In total, the number of larviposition was approximately 90-100 in viviparous females [95.25 ± 24.75 (mean ± SD)], while lifetime oviposition by oviparous females was less than one-third of larviposition by viviparous females [28.83 ± 6.52 (mean ± SD)]. Cross marks indicate the death of the insect. Viviparous females and oviparous females were maintained on the leaves of bean plants, *Vicia faba*, under 16 °C, 16 h light (L):8 h dark (D) and 15 °C, 8L:16D, respectively. Oviparous females were maintained with adult males (these males were removed after their death). Viviparous females started reproduction from days 2-3 and the rate of larviposition was at the peak during days 3-20 but slow downed during days 21-28. They lived at most 50-55 days, although most of them stopped larviposition after day 30. In oviparous females, first oviposition and mating with males were observed at days 3-4. They laid eggs actively until around day 14 but their death was observed almost simultaneously.


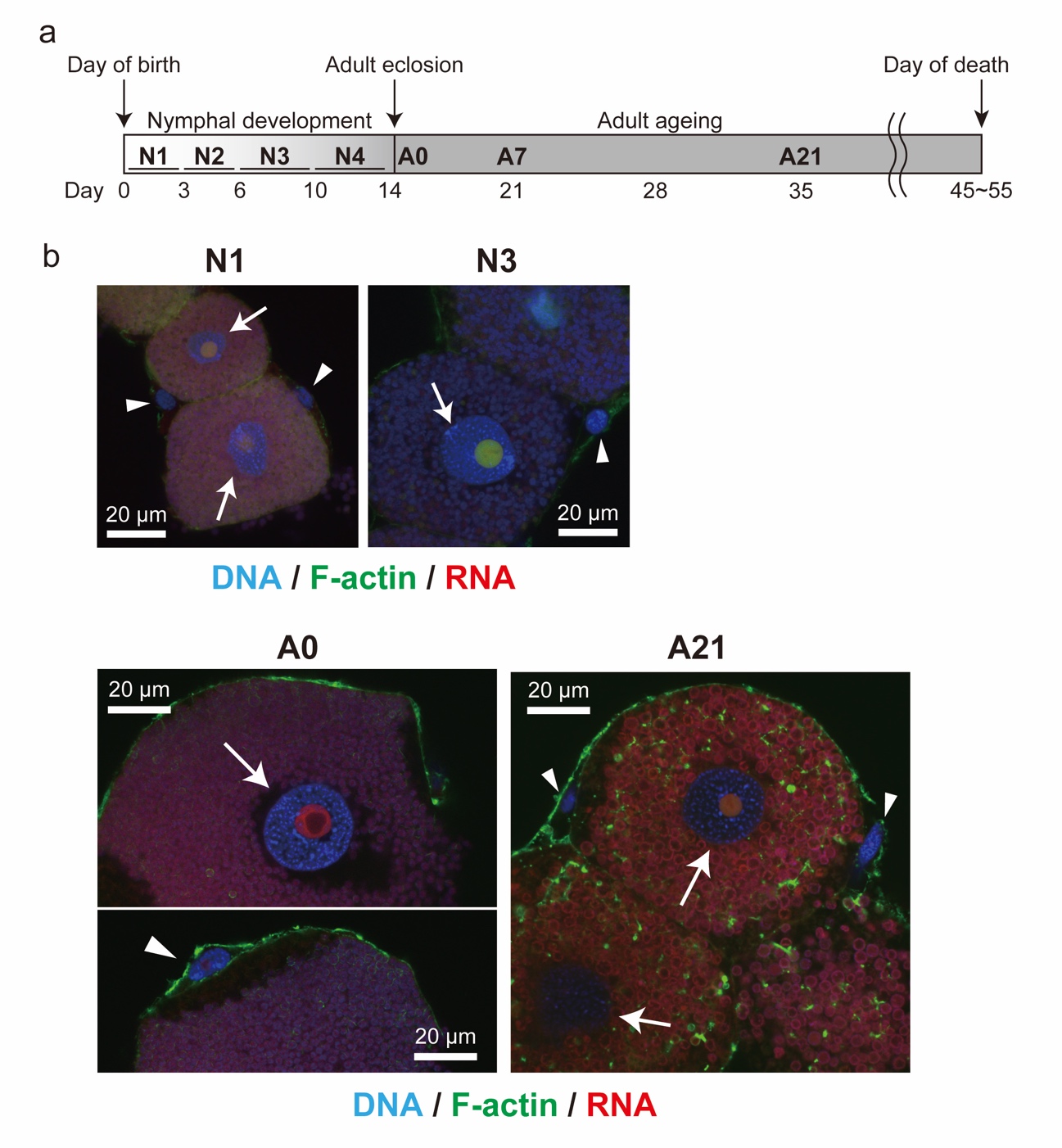


**Figure S8.** Developmental scheme and morphological changes of bacteriocytes during post-embryonic development in viviparous females. **a** First-instar nymphs of viviparous aphids molt four times during 13.73 ± 0.32 (mean ± SEM, *n* = 16) days at 16 °C. After adult eclosion, aphids live approximately 1 month under 16 °C. N1-4 represents the first to fourth instar nymphs. A0, A7, and A21 mean adult aphids at the day of eclosion, at 7 and 21 days after eclosion, respectively. **b** Confocal microscopic images of bacteriocytes and sheath cells in each stage of viviparous females. N1 and N3 were selected and presented as representatives of nymphal stages. A0 and A21 adults were presented as young adults and senescent adults, respectively. DNA and F-actin were stained using DAPI (blue) and Phalloidin (green), and nucleolus was visualized by Nucleolus Bright Red (red). *White arrows*; nuclei of bacteriocytes, *white arrowheads*; nuclei of sheath cells.


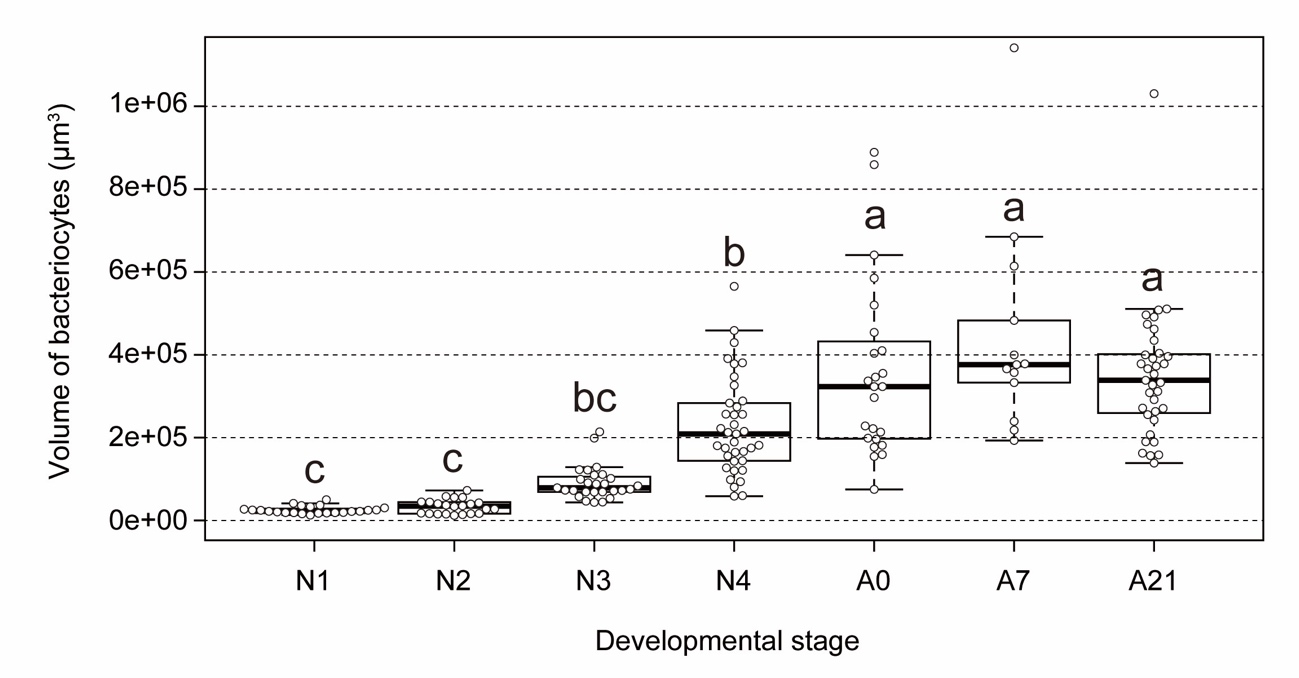
**Figure S9.** Cell volume distribution of bacteriocytes in each developmental stage of viviparous aphids. The volume of bacteriocytes was estimated based on the data from confocal microscopy (see Material and methods). Results are displayed as boxplots, where central bold lines represent the medians, boxes comprise the 25–75 percentiles, and whiskers denote the range. N1-4 represents the first to fourth instar nymphs. A0, A7, and A21 mean adult aphids at the day of eclosion, at 7 and 21 days after eclosion, respectively. The size of bacteriocytes was significantly different among developmental stages [N1; 25179.0 ± 1882.2 (mean ± SEM) μm^3^, *n* = 23, N2; 33498.2 ± 3666.5 μm^3^, *n* = 22, N3; 90773.8 ± 7859.5 μm^3^, *n* = 27, N4; 227376.6 ± 19405.5 μm^3^, *n* = 37, A0; 356132.2 ± 43503.0 μm^3^, *n* = 24, A7; 444893.4 ± 70131.0 μm^3^, *n* = 13, and A21; 350320.1 ± 27131.4 μm^3^, *n* = 35] (LMM with type II test, *p* < 0.001). Different letters indicate significant differences (Tukey’s test, *p* < 0.05).


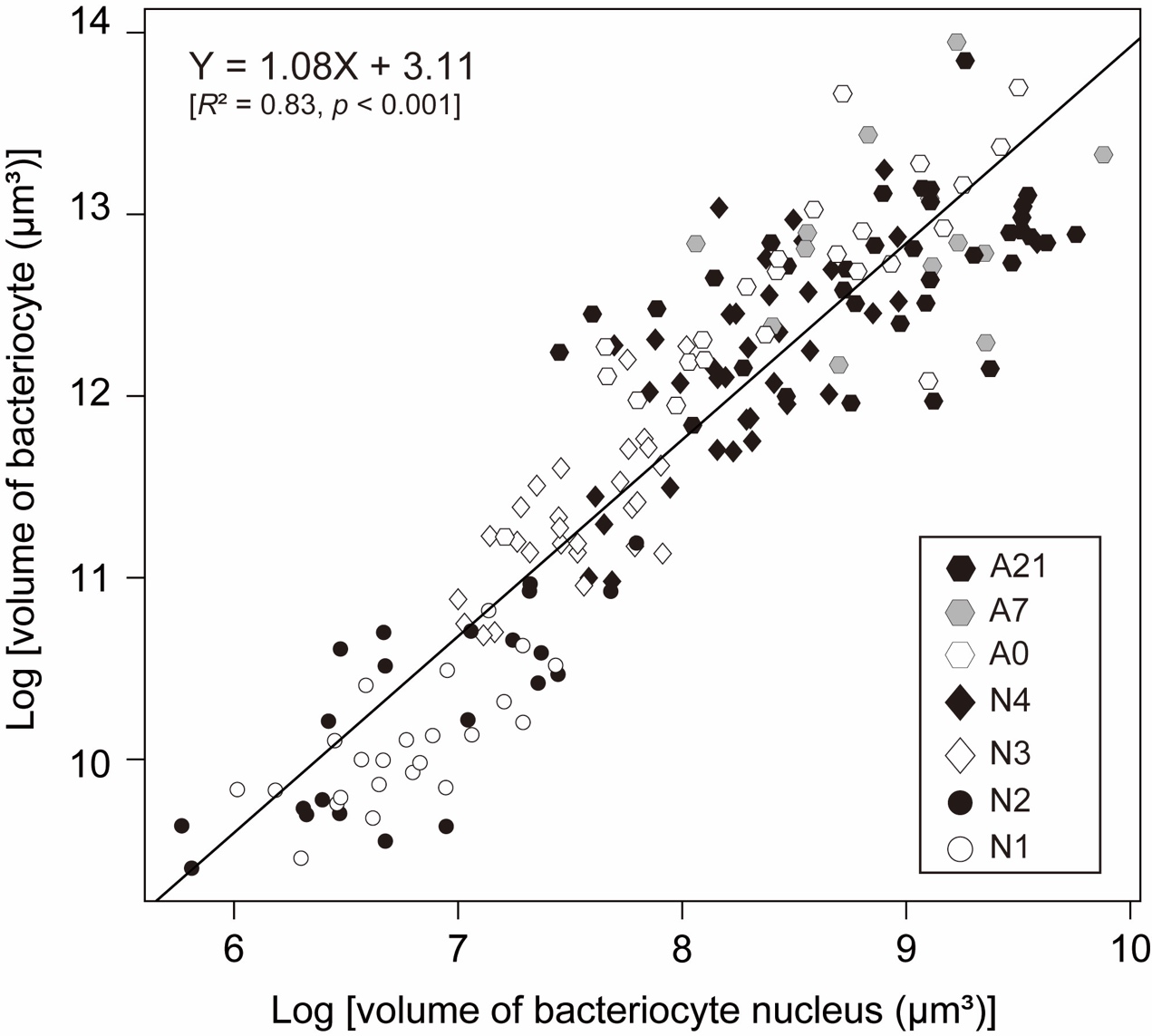


**Figure S10.** The relationship between the volume of bacteriocytes and their nuclei. The volume of bacteriocytes and their nuclei were estimated based on the data from confocal microscopy (see Material and methods). N1-4 represents the first to fourth instar nymphs. A0, A7, and A21 mean adult aphids at the day of eclosion, at 7 and 21 days after eclosion, respectively. Simple correlation analysis was conducted for the dataset from all stages of viviparous females (N1-N4 and Adult 0, Adult 7 and Adult 21 were pooled). Both parameters were log-transformed. The volume of bacteriocytes was positively correlated with those of their nuclei, which was a proxy of ploidy level (see Figure S13).


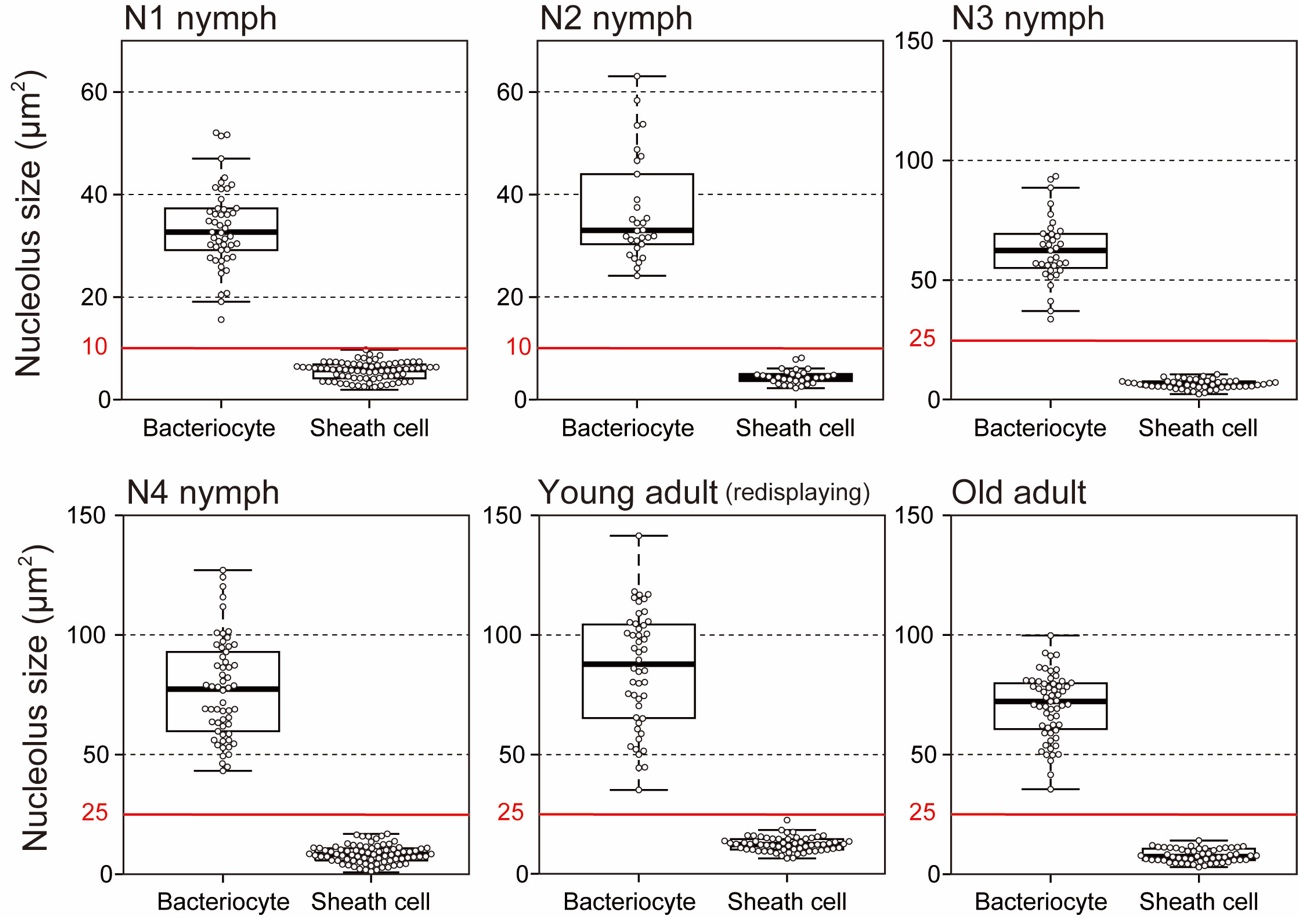


**Figure S11.** Nucleolus size distribution in each developmental stage of viviparous aphids. The size of nucleolus was significantly different between bacteriocytes and sheath cells, regardless of developmental stages (LMM with type II test, *p* < 0.001 in all). There was no overlap in nucleolus sizes between the two types of cells. N1-4 represents the first to fourth instar nymphs. A21 mean adult aphids at 21 days after eclosion. Red bars indicate the “threshold” used in our image-based fluorometry (see Material and methods). Data in the category “Young adults” was the same as “Viviparous females” in Figure S5 (re-displaying).


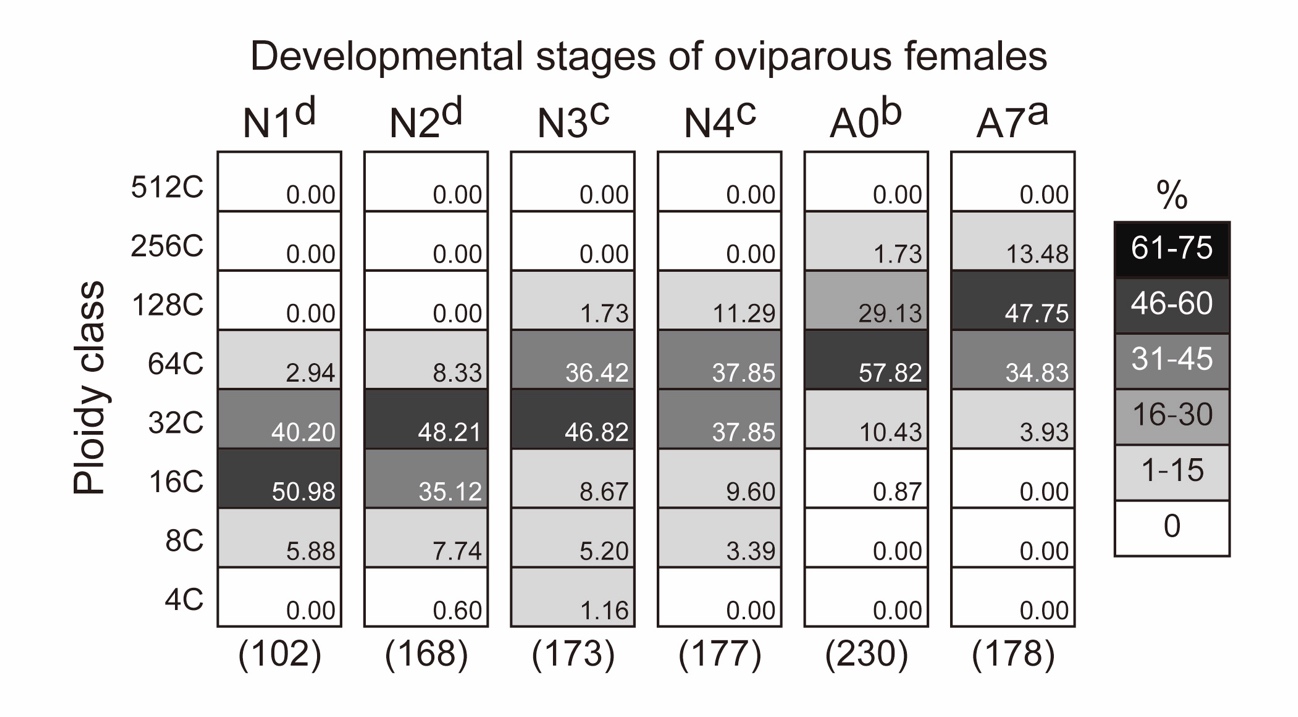


**Figure S12.** Ploidy distribution of bacteriocytes of each developmental stage of oviparous aphids. Sample size (numbers of bacteriocyte nuclei) is shown under the column. N1-4 represents the first to fourth instar nymphs. A0, A7 and A21 indicate 0-, 7-, 21-day-old adults. Different letters with aphid stages indicate significant differences in the median ploidy class (Brunner–Munzel test with Bonferroni adjustment, *p* < 0.05). Bacteriocytes of 7-day-old adults exhibited the highest polyploid level in oviparous aphids (64-128C).


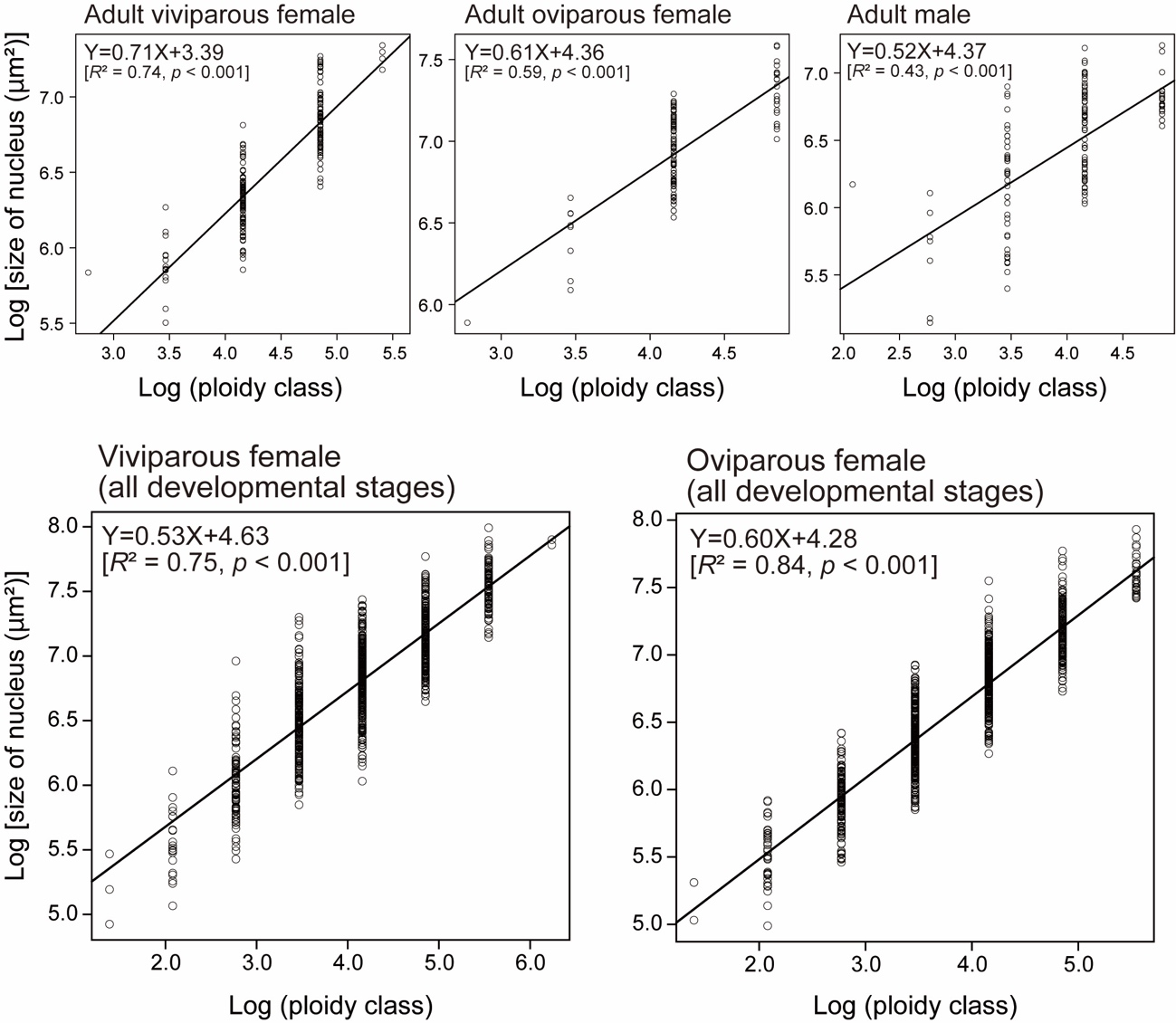


**Figure S13.** The relationship between the size (area, μm^2^) of bacteriocyte nuclei and ploidy class. Simple correlation analysis was conducted for the dataset from adult viviparous females, adult oviparous females, adult males, and all stages of viviparous/oviparous females (N1-N4 and A0, A7, A21 were pooled). Both parameters were log-transformed. The size of bacteriocyte nuclei was positively correlated with the ploidy class in all categories. In the dataset of viviparous/oviparous females that contain seven developmental stages, the simple linear correlation models were well-fitted (*R*^2^ = 0.75 and 0.84, respectively).

**References (only referred to in the SI)**

1. Sutherland, O. R. W. (1969). The role of crowding in the production of winged forms by two strains of the pea aphid, *Acyrthosiphon pisum*. Journal of Insect Physiology, 15(8), 1385–1410. https://doi.org/10.1016/0022-1910(69)90199-1
2. Ward, S. A., & Dixon, A. F. G. (1982). Selective resorption of aphid embryos and habitat changes relative to life-span. The Journal of Animal Ecology, 859–864. https://doi.org/10.2307/4010
